# Modulation of Gut Microbiota and Metabolites by Berberine in Treating Mice With Disturbances in Glucose and Lipid Metabolism

**DOI:** 10.3389/fphar.2022.870407

**Published:** 2022-06-03

**Authors:** Xinyi Fang, Haoran Wu, Xinmiao Wang, Fengmei Lian, Min Li, Runyu Miao, Jiahua Wei, Jiaxing Tian

**Affiliations:** ^1^ Department of Endocrinology, Guang’anmen Hospital, China Academy of Chinese Medical Sciences, Beijing, China; ^2^ Graduate College, Beijing University of Chinese Medicine, Beijing, China; ^3^ Graduate College, Changchun University of Chinese Medicine, Changchun, China

**Keywords:** glucose and lipid metabolism disturbances, berberine, gut microbiota, metagenomics, metabolomics

## Abstract

**Introduction:** Glucose and lipid metabolism disturbances has become the third major disease after cancer and cardio-cerebrovascular diseases. Emerging evidence shows that berberine can effectively intervene glucose and lipid metabolism disturbances, but the underlying mechanisms of this remain unclear. To investigate this issue, we performed metagenomic and metabolomic analysis in a group of normal mice (the NC group), mice with disturbances in glucose and lipid metabolism (the MC group) and mice with disturbances in glucose and lipid metabolism after berberine intervention (the BER group).

**Result:** Firstly, analysis of the clinical indicators revealed that berberine significantly improved the blood glucose and blood lipid of the host. The fasting blood glucose level decreased by approximately 30% in the BER group after 8 weeks and the oral glucose tolerance test showed that the blood glucose level of the BER group was lower than that of the MC group at any time. Besides, berberine significantly reduced body weight, total plasma cholesterol and triglyceride. Secondly, compared to the NC group, we found dramatically decreased microbial richness and diversity in the MC group and BER group. Thirdly, LDA effect size suggested that berberine significantly altered the overall gut microbiota structure and enriched many bacteria, including *Akkermansia* (*p* < 0.01), *Eubacterium* (*p* < 0.01) and *Ruminococcus* (*p* < 0.01). Fourthly, the metabolomic analysis suggested that there were significant differences in the metabolomics signature of each group. For example, isoleucine (*p* < 0.01), phenylalanine (*p* < 0.05), and arbutin (*p* < 0.05) significantly increased in the MC group, and berberine intervention significantly reduced them. The arbutin content in the BER group was even lower than that in the NC group. Fifthly, by combined analysis of metagenomics and metabolomics, we observed that there were significantly negative correlations between the reduced faecal metabolites (e.g., arbutin) in the BER group and the enriched gut microbiota (e.g., *Eubacterium* and *Ruminococcus*) (*p* < 0.05)*.* Finally, the correlation analysis between gut microbiota and clinical indices indicated that the bacteria (e.g., *Eubacterium*) enriched in the BER group were negatively associated with the above-mentioned clinical indices (*p* < 0.05).

**Conclusion:** Overall, our results describe that the changes of gut microbiota and metabolites are associated with berberine improving glucose and lipid metabolism disturbances.

## Introduction

Along with dietary habits and lifestyle modifications, the incidence of many diseases related to glucose and lipid metabolism disturbances, including obesity, hyperlipidemia, non-alcoholic fatty liver disease, type 2 diabetes, and associated cardiovascular complications associated with it, has reached epidemic levels. It is reported that about 1.5 billion people worldwide suffer from metabolic syndrome yearly, which has become a global public health concern (Maifeld et al., 2021).

In recent decades, the potential role of the gut microbiota in altering host metabolism of the hosts has drawn considerable attention. The gut microbiota affects the occurrence and development of metabolic diseases through energy metabolism, immune regulation and inflammatory mechanisms (Maruvada et al., 2017). Extensive research has shown that abnormal diet structure will leads to gut microbiota dysbiosis, leading to the production of short-chain fatty acids (SCFAs), amino-acid-derived-metabolites, and microbial-derived neurotransmitters, resulting in reduced insulin secretion, elevated blood glucose levels, metabolic endotoxemia, and finally metabolic diseases (Fan and Pedersen, 2021). It has previously been observed that, in obese individuals, there are obvious changes in gut microbiota, such as the *Mollocutes* class of the *Firmicutes*, which were significantly increased, and *Akkermansia*, *Faecalibacterium*, *Oscillibacter*, and *Alistipes*, which significantly decreased (Wu et al., 2021). It has also been found that *Bacteroides caccae*, *Escherichia coli*, *Desulfovibrio*, *Lactobacillus gasseri*, *Streptococcus mutans*, and *Hemophilus parainfluenzae* are associated with insulin resistance or type 2 diabetes, wheras *Clostridium*, *Eubacterium rectale*, *Roseburia*, *Verrucomicrobiaceae*, and *Faecalibacterium prausnitzii* have antidiabetic effects (Hartstra et al., 2015). More importantly, through faecal microbiota transplants, the gut microbiota has been implicated in the pathogenesis of metabolic syndromes. Transplanting the gut microbiota of thin individuals into obese individuals with metabolic syndrome can improve insulin sensitivity, increase the diversity of gut microbiota and enrich the butyrate-producing bacteria, to alleviate the symptoms of obesity (Vrieze et al., 2012).

Additionally, whether the metabolites can play a regulatory role in glucose and lipid metabolism disturbances has also attracted the attention of researchers. Recently, studies have found that human samples with metabolic disturbances show dysregulation of lipolysis, fatty acid oxidation, amino acid and ketone production, as well as changes in triglyceride, phospholipid and trimethylamine oxide levels (Zhang et al., 2015a; Org et al., 2017; Fiamoncini et al., 2018; Surowiec et al., 2019). The end-product of microbial fermentation, SCFAs (such as butyric acid, propionic acid, and acetic acid), can increase satiety and browning of white adipose tissue, reduce fat production, and participate in the maintenance of intestinal barrier function. Branched-chain amino acids (BCAAs), such as valine, isoleucine, and leucine, can increase heat production and protein synthesis, which is related to insulin resistance and deposition of fatty material in the vasculature. Disorder of bile acid metabolism is associated with fatty degeneration of the liver and disturbances in glucose and lipid metabolism. Tryptophan metabolism disorder is related to inflammatory pathogenesis of the liver, fatty degeneration of the liver, and insulin resistance (Agus et al., 2021). Thus, we speculate that the regulation of the gut microbiota (including its structure, composition, and function) and metabolites may provide a new strategy for the treatment of glucose and lipid metabolism disturbances.

Studies have shown that metformin, a first-line remedy for the treatment of diabetes, alters the gut microbiota structure of patients with glucose and lipid metabolism disturbances, such as enriching the bacteria which can produce SCFAs (Forslund et al., 2015). Compared with non-diabetic patients, diabetic patients on metformin had a higher relative abundance of *Akkermansia muciniphila*, a few gut microbiota associated with SCFAs production (including *Butyrivibrio*, *Bifidobacterium bifidum*, *Megasphaera*), and an operational taxonomic unit of *Prevotella* (de la Cuesta-Zuluaga et al., 2017). Most recently, many lines of seminal evidence, demonstrated for the first time that berberine, an active ingredient of *Coptis chinensis Franch.*, can effectively interfere with diseases such as glucose and lipid metabolism disturbances by regulating the structure of the gut microbiota and improving intestinal microecology. Berberine can improve insulin resistance and anti-obesity effects by upregulating the number of bacteria producing SCFAs and downregulating the number of bacteria producing BCAAs (Dai et al., 2011). Previous studies have shown that Gegen Qinlian decoction and its components berberine can regulate intestinal mucosal immunity and glucose and lipid metabolism, reduce systemic and islet local inflammation levels, and improve insulin resistance by enriching the bacteria producing butyric acid (such as *Faecalibacterium* and *Roseburia*), thus relieving type 2 diabetes mellitus. The effect of berberine on glucose metabolism compared with metformin was previously established by other authors, and we chose a single statistically significant effective dose to study the complicated gut microbial and metabolomic changes that require expensive and time-consuming analyses (Yin et al., 2002; Xu et al., 2020).

To date, limited studies have indicated a direct association between the gut microbiota and berberine intervention on glucose and lipid metabolism disturbances. Several important gaps in knowledge remain unexplored, such as less in-depth research on berberine regulation of host glucose and lipid metabolism from the perspective of gut microbiota and metabolites, and the relevant targets of drugs acting on metabolic pathways are not clear. Therefore, this study sought to observe the gut microbiota and metabolites related to glucose and lipid metabolism disturbances and berberine’s effections by metagenomic sequencing and metabolomic analysis of the NC, MC, and BER groups, and to further, probe the possible mechanism of berberine-mediated regulation metabolism by interfering with gut microbiota and metabolites in mice with disturbances in glucose and lipid metabolism.

## Results

### Effects of Berberine on Host Metabolism

**FIGURE 1:**
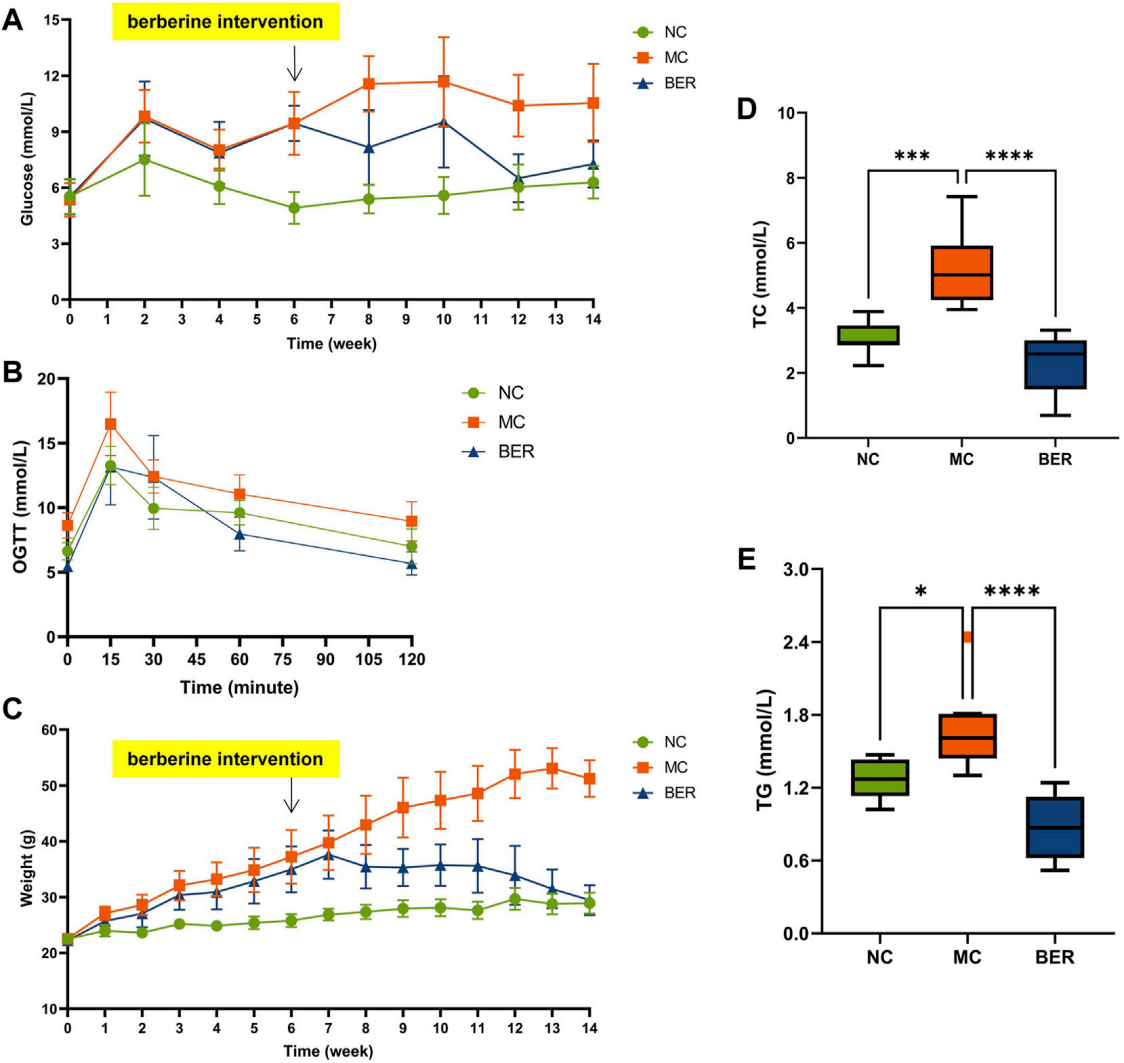
Berberine improves glucose and lipid metabolism. **(A)** fasting blood glucose (FBG). **(B)** oral glucose tolerance test (OGTT) after 14 weeks of treatment. **(C)** weight. **(D)** total plasma cholesterol (TC). **(E)** triglyceride (TG). Data are presented as means ± standard errors of the means (SEM). ****p < 0.0001, ***p < 0.001, **p < 0.01, *p < 0.05.

We analyzed clinical data from the NC, MC, and BER groups, as shown in Supplementary Table S1. The baseline variables showed no significant differences among the three groups. The fasting blood glucose level decreased by approximately 30% in the BER group compared with that in the MC group after 8 weeks. At 12 weeks, the fasting blood glucose level in the BER group were very close to those in the NC group (Figure 1A). The oral glucose tolerance test showed that the blood glucose level of the BER group was lower than that of the MC group at any time and even after 60 min the value was lower than that of the NC group (Figure 1B). In addition, berberine significantly reduced the weight of mice with disturbances in glucose and lipid metabolism at 7 weeks. The weight of mice in the BER group was similar to the NC group at 14 weeks of intervention. (Figure 1C). It is worth noting that berberine significantly reduced total plasma cholesterol (TC) and triglyceride (TG) (*p* < 0.0001), which were, lower than those in the NC group (Figures 1D,E). However, there were no significant differences between the three groups in high-density lipoprotein and low-density lipoprotein levels (Supplementary Figures S1A,B).

This is a rather significant result indicating that berberine can effectively reduce host blood glucose, TC, TG, and weight loss.

### Structural Changes of Gut Microbiota in Each Group

**FIGURE 2:**
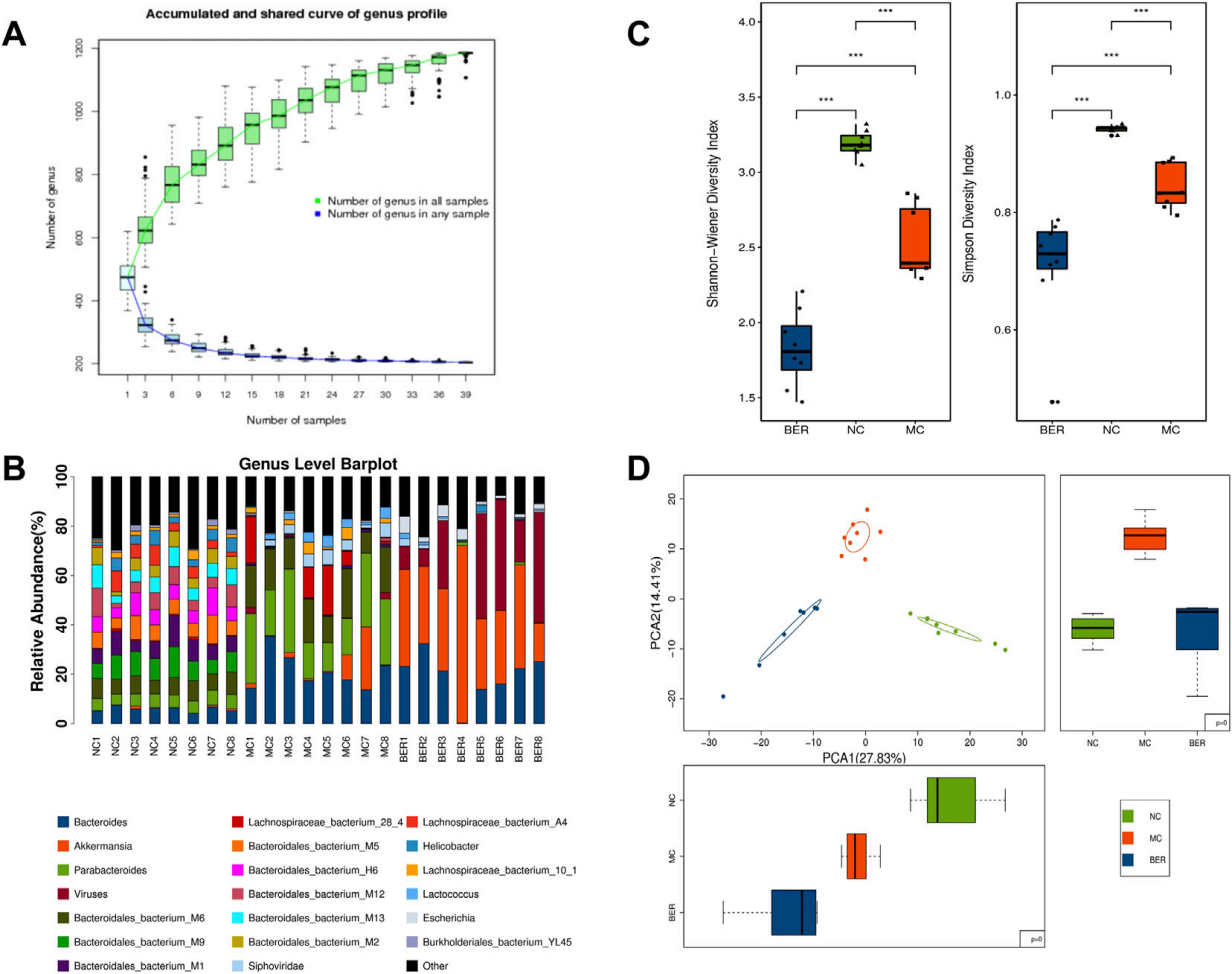
Decreased diversity and gut microbiota structure altered in the MC group and BER group. **(A)** The species accumulation curves. The abscissa represents the number of samples, and the ordinate represents the number of species after sampling. **(B)** The genus level of the relative abundance barplot. It shows the top 20 species composition of each sample, while the sum of the abundance of species other than the top 20 is represented in black. **(C)** Box chart of the Shannon-Wiener index and Simpson index. *0.01 < *p* < 0.05; **p < 0.01. **(D)** Principal-component analysis plot.

The abundance and composition of the gut microbiota were observed by metagenomic sequencing of mice faecal samples to explore the characteristics of the gut microbiota in mice with disturbances in glucose and lipid metabolism and changes in the gut microbiota after berberine intervention. The species accumulation curves show that within a certain range, with a gradual increase in samples, more gut microbiota species can be found, and the curves show an upward trend. Subsequently, the curve was neariy smooth, indicating that the samples were sufficient to accurately reflect the species composition of the bacterial community (Figure 2A). The genus level of the relative abundance bar plot intuitively shows significant differences in gut microbiota composition among the NC, MC, and BER groups (Figure 2B).

Species diversity analysis was performed to characterize the bacterial richness. The median and quartile of the Shannon-Weiner and Simpson indices showed that the *α* diversity at the genus level was much lower in the MC and BER groups (NC vs. MC, *p* < 0.01; NC vs. BER, *p* < 0.01), and the *α* diversity of gut microbiota in the BER group decreased significantly compared with the MC group (BER vs. MC, *p* < 0.01) (Supplementary Table S2, Figure 2C). To determine whether berberine affects the gut microbiota of mice with disturbances in glucose and lipid metabolism, we carried out a principal component analysis based on the bacterial abundance of the gut microbiota. As expected, the results showed that the gut microbiota in the NC, MC, and BER groups had an obvious clustering trend (Figure 2D). Compared with the NC group, there were significant changes in the structure of the gut microbiota in the MC group, and there were also striking differences in the structure of the gut microbiota between rhe MC and BER groups.

Collectively, these results support our hypothesis that berberine plays an important role in gut microbiota remodelling induced by disturbances in glucose and lipid metabolism.

### Locating the Key Gut Microbiota of Berberine in the Intervention of Glucose and Lipid Metabolism Disturbances

**FIGURE 3:**
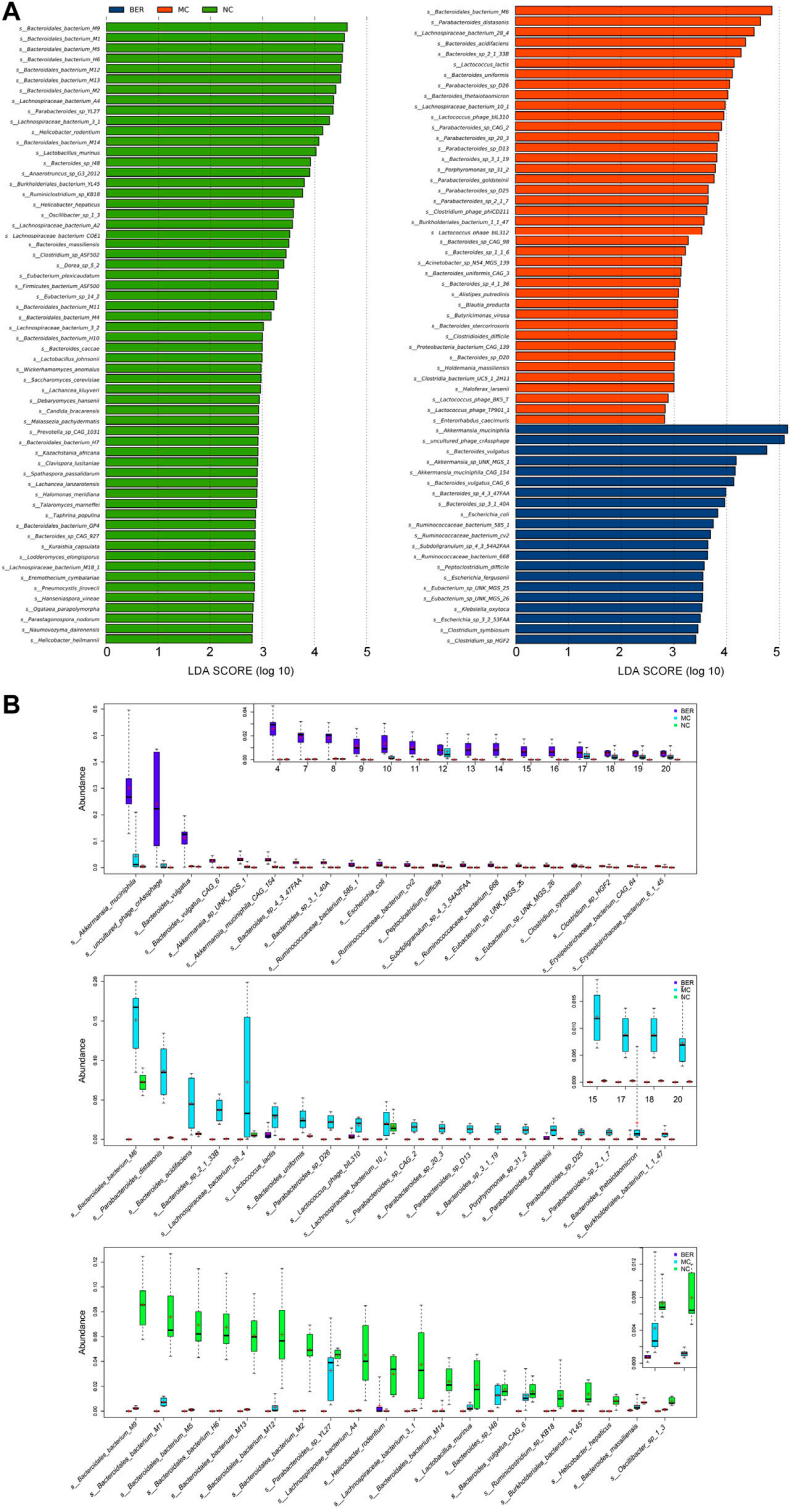
Abundance of differential species in each group. **(A)** LEfSe differential species plot. **(B)** Relative abundance box plot of dominant species in each group. Boxes represent the interquartile ranges, lines inside the boxes denote medians, and signs indicate mean value.

To further explore the differences in bacteria species, LEfSe (LDA Effect Size) software was used to analyze the relative abundance of gut microbiota between the NC, MC, and BER groups and to locate the key gut microbiota of berberine in the intervention of glucose and lipid metabolism disturbances (Figure 3A). We observed that the relative abundance of some bacteria in the NC group was higher than that in the other groups (LEfSe: LDA>2.5), mainly *Bacteroides* (e.g., *s_Bacteroidales_bacterium_M9*, *s_Bacteroidales_bacterium_M1*, and *s_Bacteroidales_bacterium_M5*), *Lactobacillus* (e.g., *Lactobacillus murinus* and *Lactobacillus johnsonii*), and *Prevotella* (e.g., *s_Prevotella_sp_CAG_1031*). In the MC group, *Lactococcus* (e.g., *Lactococcus lactis* and *s_Lactococcus_phage_bIL310*), *Porphyromonas* (e.g., *s_Porphyromonas_sp_31_2*), *Holdemania* (e.g., *s_Holdemania_massiliensis*), and *Enterorhabdus* (e.g., *s_Enterorhabdus_caecimuris*) were more abundant (LEfSe: LDA>2.5). *Akkermansia* (e.g., *Akkermansia muciniphila*, *s_Akkermansia_sp_UNK_MGS_1*,and *s_Akkermansia_muciniphila_ CAG_154*), *Eubacterium* (e.g., *s_Eubacterium_sp_UNK_MGS_25* and *s_Eubacterium_sp_UNK_MGS_26*), and *Ruminococcus* (e.g., *s_Ruminococcaceae_bacterium_585_*1, *s_Ruminococcaceae_ bacterium_cv2*, and *s_Ruminococcaceae_bacterium_668*), were enriched in the BER group (LEfSe: LDA>3).

The relative abundances of the top 20 species enriched in the BER, MC, and NC groups are shown in Figure 3B. As shown in the figure, *s_Bacteroidales_bacterium_M9* (*p* < 0.01), *s_Bacteroidales_bacterium_M1* (*p* < 0.01), *s_Bacteroidales_bacterium_M5* (*p* < 0.01), and *Lactobacillus murinus* (*p* < 0.01) were enriched in the NC group. Compared with the NC and BER groups, *Lactococcus lactis* (*p* < 0.01) and *s_Lactococcus_phage_bIL310* (*p* < 0.01) were enriched in the MC group. Moreover, in contrast to the NC and MC groups, the abundance of gut microbiota such as *Akkermansia muciniphila* (*p* < 0.01), *s_Akkermansia_sp_UNK_MGS_1* (*p*<0.01), *s_Akkermansia_muciniphila_CAG_154*(*p*<0.01), *s_Eubacterium _sp_UNK_MGS_25*(*p*<0.01), *s_Eubacterium_sp_UNK_MGS_26* (*p* < 0.01), *s_Ruminococcaceae_bacterium_585_1* (*p* < 0.01), *s_Ruminococcaceae_bacterium_cv2* (*p* < 0.01), and *s_Ruminococcaceae_bacterium_668* (*p* < 0.01) in the BER group was significantly increased.

Altogether, these results indicate that an increase in *Lactococcus*, *Porphyromonas*, *Holdemania*, and *Enterorhabdus* is the most important feature of gut microbiota dysbiosis in mice with disturbances in glucose and lipid metabolism. In addition, these findings highlighted the possibility of *Akkermansia*, *Eubacterium*, and *Ruminococcus* as the key genera associated with berberine’s interference with glucose and lipid metabolism disturbances.

### Functional Alteration in the Gut Microbiota of NC, MC, and BER

**FIGURE 4:**
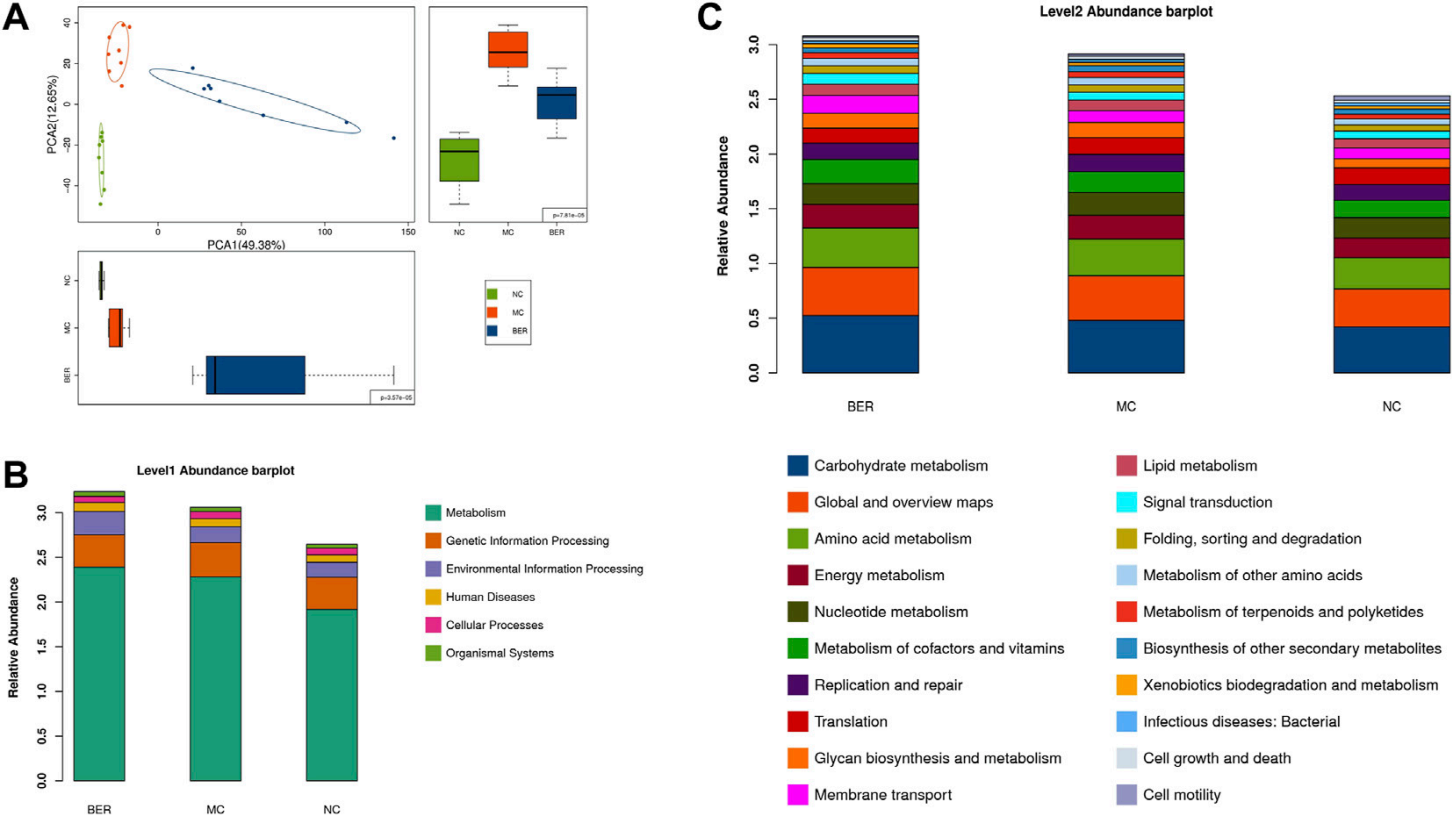
Microbial gene functions annotation in each group. **(A)** PCA based on the relative abundance of KEGG orthology groups in samples. **(B)** Abundance classification plot of level 1 in each group. **(C)** Abundance classification plot of level 2 in each group.

Changes in the gut microbiota structure are often accompanied by changes in intestinal microbial function. To clarify the functional characteristics of gut microbiota in mice with disturbances in glucose and lipid metabolism and the effect of berberine, we evaluated gut microbial functions across groups using the Kyoto Encyclopedia of Genes and Genomes (KEGG) database. Principal component analysis (PCA) based on KEGG orthology revealed striking differences in microbial functions in the first principal component among the NC, MC, and BER groups (Figure 4A). We observed that compared with the NC group, the gut microbiota function of mice with disturbances in glucose and lipid metabolism had significant changes, and berberine could affect it (Supplementary Figure S1C). The KEGG level 1 pathway analysis revealed BER-induced changes (Figure 4B). The relative abundances of KOs associated with metabolism and environmental information processing significantly increased after the model mice were fed berberine. The KEGG level 2 bar plot showed that compared with the NC group, bacterial genes associated with glucose, lipid, amino acid and energy metabolisms, metabolism of cofactors and vitamins, and glycan biosynthesis and metabolism increased significantly in the MC group, whereas bacterial genes associated with cell motility were significantly reduced. In addition, compared with the MC group, bacterial genes associated with glucose, lipid, and amino acid metabolisms, membrane transport, and signal transduction were significantly increased after berberine intervention, whereas those associated with the biosynthesis of other secondary metabolites were significantly decreased (Figure 4C). Although functional annotation analyses are predictive, they indicate that impairment of glucose and lipid metabolism disturbances may evoke a disease-linked state by interfering with physiologic and metabolic functions.

### Effect of Berberine on Faecal Metabolomics in Mice With Disturbances in Glucose and Lipid Metabolism

Given the above, metagenomic functional annotation analysis revealed that berberine could alter the gut microbiota function of mice with disturbances in glucose and lipid metabolism. We further used gas chromatography-mass spectrometry to detect the metabolomics of faecal samples.

PCA (Supplementary Figure S2A), partial least-squares discriminant analysis (PLS-DA) (Supplementary Figure S2B) and orthogonal partial least-squares discriminant analysis (OPLS-DA) (Supplementary Figure S2C) were performed on faecal metabolites in the NC and MC groups. The compositional changes in faecal metabolites in the mice involved 202 significantly different analytes between the two groups (Supplementary Figure S2D). PCA (Supplementary Figure S2E), PLS-DA (Supplementary Figure S2F) and OPLS-DA (Supplementary Figure S2G) were performed on faecal metabolites in the NC and BER groups. The compositional changes in faecal metabolites in the mice involved 213 significantly different analytes between the two groups (Supplementary Figure S2H). PCA (Supplementary Figure S2I), PLS-DA (Supplementary Figure S2J) and OPLS-DA (Supplementary Figure S2K) were performed on faecal metabolites in the MC and BER groups. The compositional changes in faecal metabolites in the mice involved 50 significantly different analytes between the two group (Supplementary Figure S2L).

**FIGURE 5:**
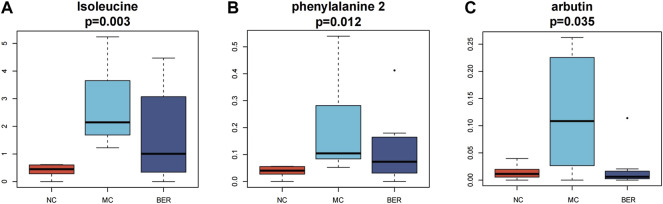
Metabolomic characteristics of the NC group, MC group and BER group. **(A,B,C)** Boxplot of differential metabolites.

Next, we refined the multigroup differential metabolite analysis (Supplementary Figure S1D). Notably, differential metabolites such as isoleucine, phenylalanine, and arbutin were the key points of our attention, because these metabolites are closely related to the gut microbiota. Accordingly, isoleucine (*p* < 0.01), phenylalanine (*p* < 0.05), and arbutin (*p* < 0.05) were significantly increased in mice with disturbances in glucose and lipid metabolism, and berberine intervention significantly reduced them. The arbutin content in the BER group was even lower than that in the NC group (Figures 5A–C).

Through metabolic pathway analysis of differential metabolites, including enrichment analysis and topological analysis of the pathway in which the differential metabolites are located, we can further screen out the pathway with the highest differential correlation with the metabolites. The differential metabolites among the MC, NC, and BER groups were significantly related to the phenylalanine, tyrosine and tryptophan biosynthesis and glycolysis/gluconeogenesis pathway.

These compounds may be derived from gut microbiota or their fermented products. To explore this idea, the relationship between metabolites and bacteria was examined by correlation analysis.

### Association Analysis Between Metagenomics and Metabolomics

To illustrate the relationship between faecal metabolites and gut microbiota in mice with disturbances in glucose and lipid metabolism, and the internal relationship between the representative metabolites and the enrichment genera after berberine intervention, the metabolomic and metagenomic data were further analyzed.

**FIGURE 6:**
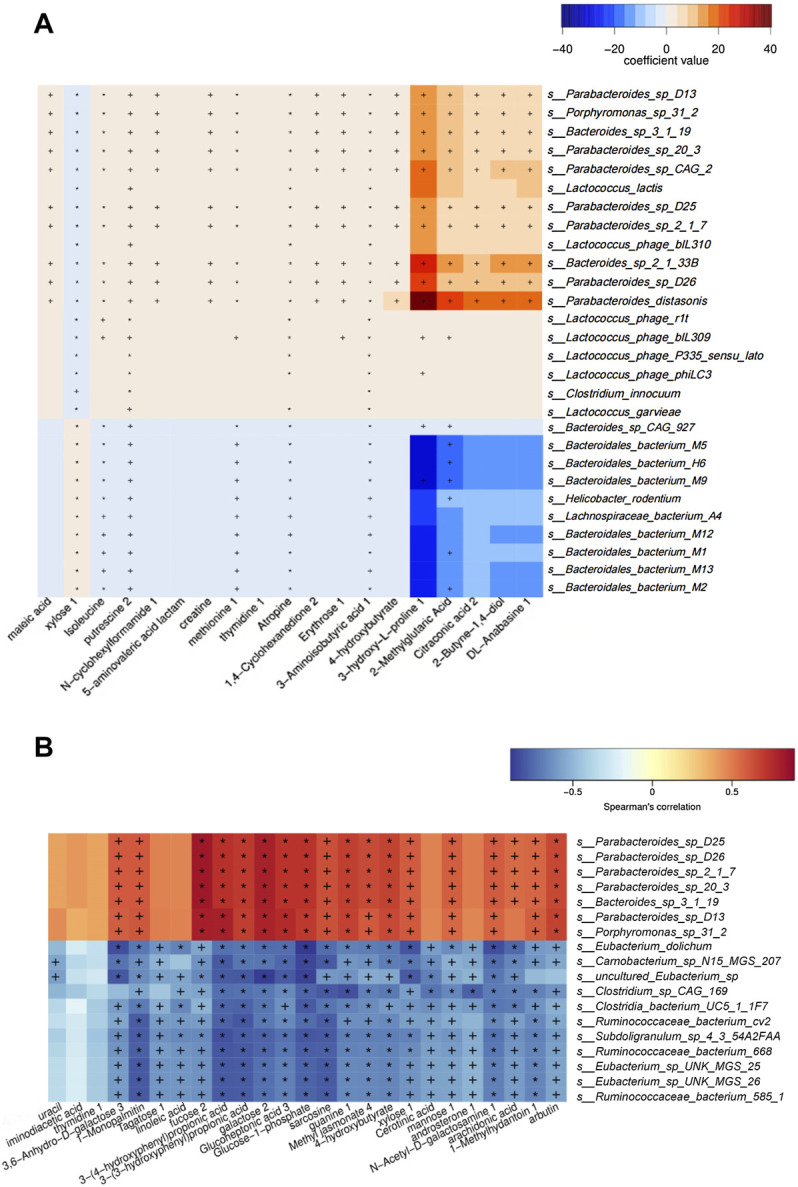
Association analysis between metagenomics and metabolomics. **(A)** Maslin analysis heat map of bacteria and metabolites (NC group vs. MC group). +q < 0.05; *q < 0.01. **(B)** Heat map of correlation between differential species and differential metabolites (BER group vs. MC group). +*p* < 0.05; *p < 0.01.

MaAsLin analysis (NC group vs. MC group) results showed that enriched isoleucine was positively correlated with *Lactococcus* (e.g., *s_Lactococcus_phage_bIL310*) (q < 0.05), and *Porphyromonas* (e.g., *s_Porphyromonas_sp_31_2*) (q < 0.01) in the MC group, whereas the metabolites were significantly negatively linked to a variety of bacteria in the NC group, such as *Bacteroides* (e.g., *s_Bacteroidales_bacterium_M9* and *s_Bacteroidales_bacterium_M5*) (q < 0.01) (Figure 6A). Additionally, in the heat map based on Spearman’s correlation analysis (MC group vs. BER group), we found that arbutin, a significantly reduced metabolite in the BER group, was negatively correlated with a variety of bacteria enriched in the BER group, such as *Eubacterium* (e.g., *s_Eubacterium_sp_UNK_MGS_25* and *s_Eubacterium_sp_UNK_MGS_26*) (*p* < 0.05) and *Ruminococcus* (e.g.,*s_Ruminococcaceae_bacterium_585_*1, *s_Ruminococcaceae_ bacterium_cv2*, and *s_Ruminococcaceae_bacterium_668*) (*p* < 0.05). In contrast, the above metabolites were significantly positively linked to the bacteria enriched in the MC group, such as *Porphyromonas* (e.g., *s_Porphyromonas_sp_31_2*) (*p* < 0.01) (Figure 6B).

Collectively, these findings provide novel evidence that the changes in faecal metabolites in mice with disturbances in glucose and lipid metabolism were significantly related to the changes in the gut microbiota, and there was a correlation between specific faecal metabolites and the enrichment of gut microbiota in mice with disturbances in glucose and lipid metabolism after berberine intervention.

### Associations of Gut Microbial Species With Clinical Indices

**FIGURE 7:**
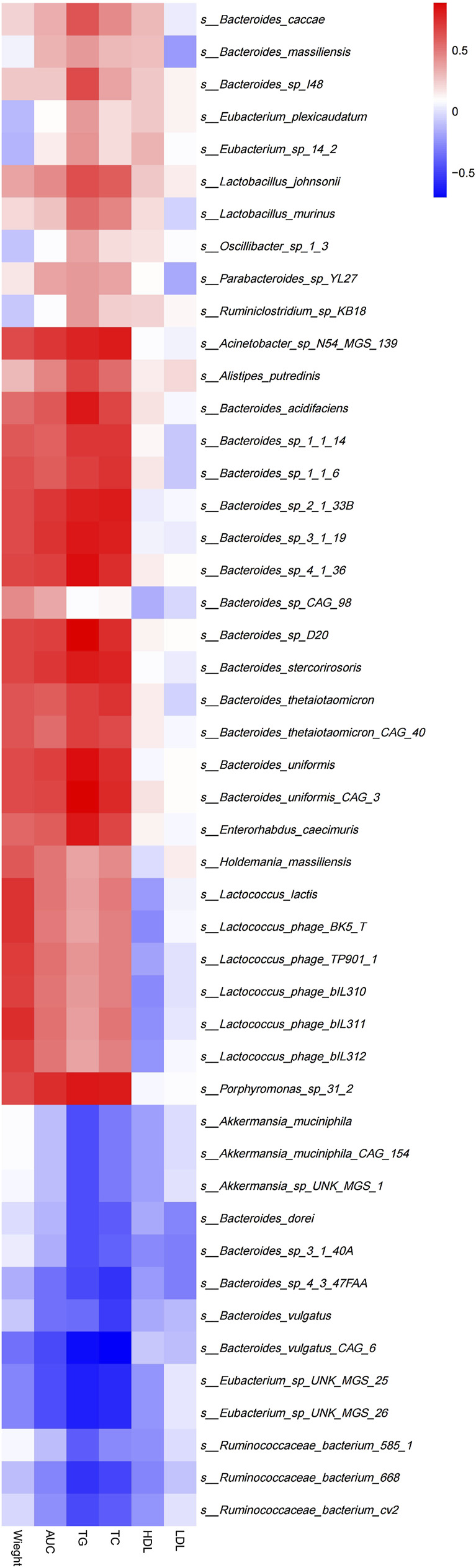
Correlation between structural characteristics of gut microbiota and clinical characteristics in each group. AUC, area under the curve (AUC) during OGTT; TG, triglycerides; TC, total cholesterol; LDL, low-density lipoprotein; HDL, high-density lipoprotein.

Spearman’s correlation analysis was used to explore the correlation between gut microbiota composition and clinical features in mice (Figure 7). The results demonstrated that *Lactococcus*(e.g.,*Lactococcuslactis* and *s_Lactococcus_phage_ bIL310*)(*p*<0.05),*Porphyromonas*(e.g., *s_Porphyromonas_sp_31_ 2*) (*p* < 0.01), *Holdemania* (e.g., *s_Holdemania_massiliensis*) (*p* < 0.05) and *Enterhabdus* (e.g., *s_Enterorhabdus_caecimuris*) (*p* < 0.01) that were significantly enriched in the MC group were positively correlated with body weight and the area under the curve during oral glucose tolerance test, TG, TC, and *Eubacterium*(e.g.,*s_Eubacterium_sp_UNK_MGS_*25, *s_Eubacterium_ sp_UNK_MGS_26*) (*p* < 0.05) that were significantly enriched in the BER group were negatively correlated with the area under the curve during oral glucose tolerance test, TG, TC and body weight.

## Discussion

The conditions used in this study showed similar results to dose-response studies, which indicated that berberine has hypoglycemic and lipid-lowering effects. The blood glucose, TC, TG, and bodyweight of the mice decreased after berberine intervention. Our results indicated that the beneficial effects of berberine on glucose and lipid metabolism disturbances were associated with changes in the gut microbiota and metabolites. Based on metabolomic and metagenomic analyses, we observed significant dysbiosis of gut microbiota in the MC group. Berberine could improve glucose and lipid metabolism disturbances by regulating gut microbiota and metabolites. Firstly, it seems that at the genus level, the α-diversity of the MC and BER groups was significantly lower than that of the NC group, which means that glucose and lipid metabolism disturbances can reduce the diversity of gut microbiota. Meanwhile, compared with the MC group, the species diversity of the gut microbiota in the BER group decreased significantly. It is worth mentioning that berberine significantly downregulated the diversity and abundance of the gut microbiota in mice with disturbances in glucose and lipid metabolism, which may be explained by the fact that it is a natural antibacterial agent (Han et al., 2020) and inhibits bacteria associated with diseases. Our finding, berberine reduced gut microbiota diversity, is consistent with previous reaserches (Zhang et al., 2015b; Xu et al., 2017). The PCA and the relative abundance bar plot of gut microbiota indicated that the composition of gut microbiota in each group was significantly different, implying that berberine could not completely reverse the gut microbiota structure in mice with disturbances in glucose and lipid metabolism in the current study, which may be related to the short intervention period, or the inhibition of diseases related bacteria. In particular, based on LEfSe differential analysis of species, we observed that the increasein *Lactococcus*, *Porphyromonas*, *Holdemania*, and *Enterorhabdus* was the most important feature of gut microbiota in the MC group, and *Akkermansia*, *Eubacterium*, and *Ruminococcus* were the three pivotal genera of berberine that improved glucose and lipid metabolism disturbances. Concomitant with the alteration of the gut microbial composition, we observed a dysbiosis in bacterial gene functions. Functional differences in the gut microbiota between the NC, MC, and BER groups mainly existed in glucose, lipid, and amino acid metabolisms. Moreover, based on metabolomic analysis and its combined analysis with metagenomic, we found that the faecal metabolomic characteristics in the MC and BER groups changed significantly, and the association between the representative metabolites and specific bacteria should not be ignored. The metabolomic analysis results suggested that the contents of isoleucine, phenylalanine, and arbutin in the MC group were upregulated. The three metabolites, particularly arbutin, were significantly downregulated after berberine intervention. The arbutin content of the BER group was lower than that of the NC group. Correlation analysis showed that the level of isoleucine was positively correlated with *Lactococcus* and *Porphyromonas* in the MC group, and negatively correlated with *Bacteroides* in the gut microbiota of the NC group. The level of arbutin was negatively correlated with *Eubacterium* and *Ruminococcus* in the BER group, and positively correlated with *Porphyromonas* in the gut microbiota of the MC group. Finally, one of the most significant findings of this study is that the bacteria enriched in the MC group (e.g., *Lactococcus*, *Porphyromonas*, *Holdemania*, and *Enterhabdus*) were positively correlated with body weight, blood glucose, TG and TC. The bacteria enriched in the BER group (e.g., *Eubacterium*) were negatively correlated with the above-mentioned clinical indices.

By analyzing the metagenomic results, we found that the relative abundance of gut microbiota such as *Bacteroides*, *Lactobacillus*, and *Prevotella* in the NC group was higher than that in the other groups. Consistent with the results of this study, previous studies have revealed that the enrichment of these three bacteria has a protective effect against glucose and lipid metabolism disturbances. Genera such as *Akkermansia* and *Clostridiales* were overrepresented in individuals with type 2 diabetes mellitus, whereas *Bacteroides*, *Clostridium*, and *Prevotella* decreased (Shapiro et al., 2018). Additionally, recent studies have found that *Lactobacillus*, as a probiotic, has a strong glycolysis ability (Newman et al., 2020; Rodrigues et al., 2021). Accumulating evidence suggests that the gut microbiota contributes to the development of glucose and lipid metabolism disturbances. Thus, characterization of the gut microbiota in glucose and lipid metabolism disturbances and identification of microbial therapeutic targets are warranted. We found that the increase in *Lactococcus*, *Porphyromonas*, *Holdemania*, and *Enterrhabdus* is the most important feature of the gut microbiota in mice with disturbances in glucose and lipid metabolism. Previous studies have suggested that these four genera are closely related to disturbances in glucose and lipid metabolism. For example, *Streptococcus sp.* and *Lactococcus sp.* are abundant in the gut microbiota of infants with type 1 diabetes mellitus (Stewart et al., 2018). It was originally reported that compared with the control group, participants who developed type 2 diabetes had a higher abundance of *Roseburia hominis*, *Porphyromonas bennonis* and unclassified *Paraprevotella* (Wang et al., 2021), and it has been reported that the *Holdemanella* genus showed a positive association with the android fat ratio in men (Min et al., 2019). Moreover, a previous study reported that the relative abundance of *Enterhabdus* was significantly positively correlated with obesity (Li et al., 2019). Our results are consistent with those of previous studies, indicating that glucose and lipid metabolism disturbances may be related to the enrichment of *Lactococcus*, *Porphyromonas*, *Holdemania*, and *Enterhabdus.*

Notably, our study provides direct evidence that berberine can improve gut microbiota dysbiosis in mice with disturbances in glucose and lipid metabolism. As shown by our data, *Akkermansia*, *Eubacterium*, and *Ruminococcus* in mice with disturbances in glucose and lipid metabolism increased significantly after berberine intervention, indicating that these genera may be involved in berberine’s improvement of glucose and lipid metabolism disturbances. It has been documented that *Ruminococcus* is an important acetate-producing bacteria in the gut (Moreno-Navarrete et al., 2018). *Akkermansia* is considered a new biomarker of intestinal health and can improve obesity and glucose homeostasis by secreting glucagon-like peptide-1-inducing protein (Yoon et al., 2021). Until now, *Akkermansia* is associated with a reducion in intestinal inflammation, improvement in impaired glucose tolerance, dyslipidemia and insulin resistance, and alleviation of type 2 diabetes mellitus (Bodogai et al., 2018; Foley et al., 2018; Nie et al., 2019; Gurung et al., 2020). In agreement with our findings, previous studies have revealed that berberine can significantly improve obesity and diabetes by enriching *Akkermansia* and *Ruminococcus*. Evidence suggests that both dihydroberberine and berberine can enrich *Akkermansia* (Li et al., 2020). Berberine reduced the richness and diversity of gut microbiota but increased the abundance of beneficial genera such as *Bacteroides*, *Bifidobacteria*, *Lactobacillus*, and *Akkermansia* (Fang et al., 2021). Researchers have also shown that berberine intervention can increase the abundance of *Ruminococcus* and improve obesity and inflammatory responses induced by a high-fat diet (Zhang et al., 2012). Earlier studies have shown that *Eubacterium* is a representative genus that produces butyrate, which can alleviate intestinal inflammation, type 2 diabetes mellitus and obesity symptoms by producing SCFAs (Louis et al., 2014; Mukherjee et al., 2020). However, whether berberine improve glucose and lipid metabolism disturbances is associated with the enrichment of *Eubacterium* in the gut requires further in‐depth verification.

Analysis of faecal metabolomics revealed that isoleucine, phenylalanine and arbutin levels increased significantly in the MC group. Further studies are warranted to delineate phenylalanine, tyrosine, and tryptophan biosynthesis and glycolysis/gluconeogenesis metabolic pathways, which were the most correlated pathways of differential metabolites in mice with disturbances in glucose and lipid metabolism. Previous studies have indicated that isoleucine, a BCAA is positively correlated with insulin resistance, obesity and diabetes (Tillin et al., 2015; Gannon et al., 2018). Recent studies have shown that amino acid metabolism-related pathways involved in the biosynthesis of phenylalanine, tyrosine and tryptophan are highly enriched in obese individuals (Liu et al., 2017), and the abundance of the glycolysis/gluconeogenesis pathway is significantly increased in the gut microbiota of obese mice (Song et al., 2019). It is worth noting that the levels of faecal metabolites such as isoleucine, phenylalanine, and arbutin significantly decreased after berberine intervention. Our findings suggest that berberine’s improvement of glucose and lipid metabolism disturbances may be related to the reduced synthesis of isoleucine, phenylalanine, and arbutin, thereby regulating phenylalanine, tyrosine, tryptophan biosynthesis and glycolysis/gluconeogenesis. According to a relevant report, arbutin is a natural compound found in a number of plants, and alpha arbutin is an isomer produced by some microbial enzymes (Zhu et al., 2018). We confirmed the identity of the metabolites by LECO-Fiehn Rtx5 database information comparison, which was unable to distinguish isomers. Therefore, what we detected might be alpha arbutin. We plan to conduct further experiments in the future, using targeted metabolomics for detection and identification.

Notably, the metagenomics and metabolomics correlation analyses of our study revealed that the level of isoleucine in the MC group increased significantly, which was positively correlated with the abundance of genera enriched in the gut, such as *Lactococcus* and *Porphyromonas*. After berberine intervention, the level of arbutin in the BER group decreased, and was significantly negatively correlated with the abundance of genera enriched in the gut, such as *Eubacterium* and *Ruminococcus*. Recent studies have found that in the process of Alzheimer’s disease, dysbiosis of gut microbiota (such as an increase in the abundance of *Firmicutes* and *Verrucomicrobia*) can lead to an abnormal increase in phenylalanine and isoleucine in peripheral blood (Wang et al., 2019). *Prevotella copri* and *Bacteroides vulgatus* are involved in the synthesis and degradation of BCAAs (Pedersen et al., 2016). The increase in gut *Lactococcus* may be caused by high-fat feed pollution (Bisanz et al., 2019). *Porphyromonas gingivalis* can influence fatty acid metabolism by increasing the level of free fatty acids in mice (Wu et al., 2018). Collectively, these findings suggest that isoleucine might be related to gut microbiota such as *Firmicutes*, *Verrucomicrobia*, *Prevotella copri*, and *Bacteroides vulgatus*. *Lactococcus* might be associated with high-fat feed pollution, and *Porphyromonas* is possibly related to metabolites such as free fatty acids. However, the relationship between *Lactococcus*, *Porphyromonas* and the metabolite isoleucine has not been reported, which calls for further exploration. Lee et al. reported that primary bile acids in faeces and *Ruminococcaceae* were negatively correlated (Lee et al., 2020). An investigation found that *Eubacterium* can use lactic acid to produce butyric acid, which is conducive to the healthy and stable development of the intestinal micro-ecosystem in infants (Wopereis et al., 2018). To date, no negative correlation has been found between *Ruminococcus*, *Eubacterium* and arbutin. Further in‐depth and effective research is needed to verify and explore the results of our study.

Further analysis of the correlation between the gut microbiota and clinical indices in each group showed that the bacteria enriched in the MC group were positively correlated with body weight, blood glucose, TG, and TC. The number of bacteria enriched in the BER group was negatively correlated with these indices. This particularly encouraging finding indicated that the abnormal metabolic indicators of mice with disturbances in glucose and lipid metabolism were related to the enrichment of *Lactococcus*, *Porphyromonas*, *Holdemania*, and *Enterhabdus*, and berberine may reduce the clinical indices related to glucose and lipid metabolism by interfering with the gut microbiota in mice with disturbances in glucose and lipid metabolism (such as *Eubacterium*), to regulate host metabolism.

In summary, this is the first study to simultaneously detect the effects of berberine on the gut microbiota and metabolites. Since microbial and metabolic changes are highly significant, they are worth reporting and a full dosing follow-up study might be performed. Our results showed that berberine significantly improved the blood glucose, blood lipid and bodyweight of mice with disturbances in glucose and lipid metabolism. In this study, metagenomics and metabolomics were used to demonstrate that *Lactococcus* and *Porphyromonas* were significantly enriched in mice with disturbances in glucose and lipid metabolism, and the genera closely related metabolites such as isoleucine were found. More importantly, our study is the first to reveal that berberine reduces the production of arbutin and downregulates the glycolysis/gluconeogenesis metabolic pathway by enriching *Eubacterium* and *Ruminococcaceae*, thereby, decreasing clinical indices (body weight, blood glucose, TG, and TC). In conclusion, we clearly described the disordered profiles of gut microbiota and metabolites in mice with disturbances in glucose and lipid metabolism disturbances and analyzed the relationship between metabolomic changes and gut microbiota changes. Additionally, we provided important evidence that berberine causes gut microbial and metabolic changes which are correlated with improving the disturbances of glucose and lipid metabolism. Berberine is known as an AMP-activated protein kinase activator and can directly activate AMP-activated protein kinase which is a well-known antidiabetic mechanism (Yin et al., 2012). Notably, berberine-induced gut microbial and metabolic changes may also improve the disturbances of glucose and lipid metabolism, which need to be acknowledged and studied in the future. Further studies are needed to confirm these direct mechanistic effects. Our findings point towards a new strategy aimed at glucose and lipid metabolism disturbances interventions targeting the gut microbiota and provide new evidence for the potential mechanism of berberine in the treatment of glucose and lipid metabolism disturbances by restoring the homeostasis of gut microbiota. Follow-up research is still needed to test its causality further and explore the specific mechanisms involved.

## Methods

### Study Design

The experiments carried out in this study were approved by the animal research ethics committee of Guang’anmen Hospital. All animal care and experiments were conducted following the provisions and regulations of the national regulations on the administration of experimental animals (China, July 2013) and the regulations of Beijing Municipality on the administration of experimental animals (Beijing, China, December 2004). In this study, male C57BL/6J mice (Beijing Vital River Laboratory Animal Technology Co., Ltd., Beijing, China), weighing 20–25 g, were raised in a specific pathogen-free laboratory during the whole experiment, and they can drink water freely. Mice were placed in a restricted room, the temperature was controlled at 22–25°C, and the photoperiod was 12 h of light and 12 h of darkness. C57BL/6 J mice were fed either a sterile maintenance feed (≥18% crude protein, ≥4% crude fat, ≤5% crude fiber, and ≤8% crude ash; SPF-F02-001; SPF (Beijing) Biotechnology Co., Ltd., Beijing, China) as the NC group or high fat diet (HFD; 60% energy from fat; D12492; Research Diets, Inc., New Brunswick, NJ, United states) ad libitum for 8 weeks to induce obese diabetic mice, defined as fasting blood glucose level ≥7.0 mmol/L and gaining more than 20% body weight compared to the NC group (Aye et al., 2015; Yang et al., 2016; Son et al., 2019; Son et al., 2022). Then, mice were randomized into three groups: the NC group (*n* = 8), the MC group (*n* = 8), and the BER group (*n* = 8). Mice in the BER group raised with distilled water (the same water as the NC group and MC group) and administered with berberine (200 mg/kg). The weight of mice was measured every week. After the experiment, the mice fasted overnight, underwent cervical dislocation, and then examined the glucose and lipid metabolism.

### Stool Sample Collection and DNA Extraction

Stool samples freshly collected from each mouse were immediately frozen at −20°C, transported to the laboratory with an ice pack, and frozen in the refrigerator at −80°C. Bacterial DNA was extracted at Real Biotechnology Co., Ltd using DNA Stool Kit (Qiagen Bioinformatics Co., Ltd, Hilden, Germany) according to the manufacturer’s recommendations.

### Metagenomic Sequencing

All samples were sorted using the Illumina HiSeq 4000 platform (Illumina, Inc., United states). A paired‐end library was constructed using 500 bp as the insert of each sample. The quality of the libraries of each sample was evaluated using DNA LabChip 1000 Kit and Agilent Bioanalyzer (Agilent Technologies, United Kingdom). The Illumina raw reads were screened as follows: 1) removal of reads containing three ambiguous N bases; 2) trim the reads containing low‐quality (Q < 20) bases; and 3) delete the reads containing< 60% high‐quality bases (Phred score ≥20). Then, a SOAPaligner (version 2.21) was used for the alignment of the clean reads to the National Center for Biotechnology Information GenBank bacterial genomes.

### Microbial Relative Abundance Profiling and *de novo* Assembly

The NCBI database was used for the alignment of the clean reads for the detection of known bacteria, fungi, viruses, and archaea. The aligned reads were classified by genus and species to determine classification and abundance. The taxonomy profile was constructed at different levels. Reads were assembled using SOAPdenovo (Version 2.04). For each sample, the reads were assembled using a series of k‐mers (51, 55, 59, 63). At ambiguous Ns, the assembled scaffolds were split, and contigs above 500 bp were selected for further analysis. MetaGeneMark software (http://exon.gatech.edu/GeneMark/metagenome/Prediction/, version 3.26) was used to predicted genes. BLAST with default parameters compared the predicted open reading frames with the NCBI nonredundant sequence database.

### Gene Functional Annotation and Functional Profiling

The databases, Kyoto Encyclopedia of Genes and Genomes (KEGG), was applied for the comparison between the assembled protein sequences and the annotated gene functions. In the case of similar protein sequences (E‐value<1 × 10^−5^ and score ≥60) for another protein sequence in the database, the assembled proteins were deemed to function in line with the proteins in the database. The most effective BLAST hit was used in the analysis. Therefore, the different levels of functions were used to create the gene clusters.

### Metabolomic Analysis Based on Gas Chromatography-Mass Spectrometry

Faecal sample: transfered 50 ± 1 mg faecal sample to 2 ml EP tube, added 0.4 ml extract (methanol chloroform volume ratio = 3:1) to precipitate the protein in the sample, and added 10 μL L-2-chlorophenylalanine. Grinded the sample at 45 Hz for 4 min and centrifuged at 4°C at 12000 r/m for 15 min. Sucked 0.35 ml supernatant and transfered it to the bottle with silylated methane for subsequent detection. The instrument analysis platform was Agilent 7890 gas chromatography-mass spectrometry.

For metabolomic detection of faecal samples, the parameters of gas chromatography were as follows: Sample Volume was 1 μL, Front Inlet Mode was Splitless Mode, Front Inlet Septum Purge Flow was 3 ml min^−1^, Carrier Gas was Helium, Column was DB-5MS (30 m × 250 μm × 0.25 μm), Column Flow was 1 ml min^−1^, Oven Temperature Ramp was 80°C hold on 1 min, raised to 290°C at a rate of 10°C min^−1^, holded on 13 min, Front Injection Temperature was 280°C, Transfer Line Temperature was 295°C, Ion Source Temperature was 220°C, Electron Energy was −70 eV, Mass Range was m/z: 50–600, Acquisition Rate was 10 spectra per second, Solvent Delay was 7.9 min.

Chromatof software (V 4.3x, LECO) was used to analyze the mass spectrometry data, such as peak extraction, baseline correction, deconvolution, peak integration and peak alignment. The mass spectra and retention time indices based on metabolites were matched in the LECO-Fiehn Rtx5 database to identify metabolites.

### Statistical Analyses

All statistical analyses were performed by R software (version 3.4.2). A Nonparametric Wilcoxon test was used to determine the statistical significance of the genes, KEGG orthologs (KOs), enzymes and different taxonomic (phylum, genus, and species) levels. Determine the enrichment characteristics with a *p*-value of <0.1 after adjustment, and the determination of the enrichment group was accorded to the higher rank value. Benjamini and Hochberg’s methods were used to adjust the *p*‐values of the false discovery rate (FDR). Ade4 (Thioulouse et al., 2018) and vegan (version 2.5-1) were used for non-metric multidimensional scale (NMDS), principal coordinate analysis (PCoA), and other Multivariate community diversity analyses, while ggpubr55 (Kassambara, 2020) and ggplot2 (Wickham, 2016) were used to visualize the results. Calculation of species richness and the Shannon diversity index were conducted using the same software packages. The similarity index used was the Bray-Curtis distance matrix. Hierarchical clustering was carried out by heat map, and the distance matrix was established by Pearson correlation. The most likely explanation for the difference between the NC, MC, and BER groups (organisms or KOs) was determined through linear discriminant analysis (LDA) effect size (LEfSe) analysis.

## Data Availability

Metagenomic data has been uploaded to GSA database (No. CRA005611), https://bigd.big.ac.cn/gsa/browse/CRA005611. Once the main findings of the project have been published, the trial steering committee will review all requests for data before access is granted. If appropriate, the anonymised data and associated documentation will be made available to users under a data-sharing agreement.

## Supplementary Material

Supplementary Figure S1
**(A)** Effect of berberine intervention on high-density lipoprotein (HDL). **(B)** Effect of berberine intervention on low-density lipoprotein (LDL). Data are presented as means±standard errors of the means (SEM). *****p* <0.0001, ****p* <0.001, ***p* <0.01, **p* <0.05. **(C)** KO heat map of microbial gene functions annotation in each group. **(D)** Hierarchical cluster diagram of differential metabolites.

Supplementary Figure S2
**(A,E,I)** PCA score plots based on the metabolic profiles in faecal samples from the NC group and MC group, the NC group and BER group, the MC group and BER group. **(B,F,J)** PLS-DA score plots based on the metabolic profiles in faecal samples from the NC group and MC group, the NC group and BER group, the MC group and BER group. **(C,G,K)** Score scatter plots of OPLS-DA comparing the metabolic differences identify the separation between the NC group and MC group, the NC group and BER group, the MC group and BER group. **(D)** Metabolites significantly changed in the MC group as compared to the NC group at VIP>1 and P-value (t-test) <0.05 are identified. Venn diagrams demonstrate the number of altered metabolites shared between the NC group and the MC group by the overlap. **(H)** Metabolites significantly changed in the BER group as compared to the NC group at VIP>1 and P-value (t-test) <0.05 are identified. Venn diagrams demonstrate the number of altered metabolites shared between the NC group and the BER group by the overlap. **(L)** Metabolites significantly changed in the BER group as compared to the MC group at VIP>1 and P-value (t-test) <0.05 are identified. Venn diagrams demonstrate the number of altered metabolites shared between the MC group and the BER group by the overlap.

Supplementary Table S1 Clinical data of the NC group, MC group and BER group.

Supplementary Table S2 The median and quartile of the Shannon-Weiner index and the Simpson index in each group.

## Abbreviations

SCFAs, short-chain fatty acids; BCAAs, Branched-chain amino acids; TC, total plasma cholesterol; TG, triglyceride; KEGG, Kyoto Encyclopedia of Genes and Genomes; PCA, Principal component analysis; PLS-DA, partial least-squares discriminant analysis; OPLS-DA, orthogonal partial least-squares discriminant analysis.
