# Supplementary figures and images for: Modulation of Gut Microbiota and Metabolites by Berberine in Treating Mice With Disturbances in Glucose and Lipid Metabolism
Source: Front Pharmacol. 2022 Jun 3;13:870407. doi: 10.3389/fphar.2022.870407 (PMC9204213; doi:10.3389/fphar.2022.870407)

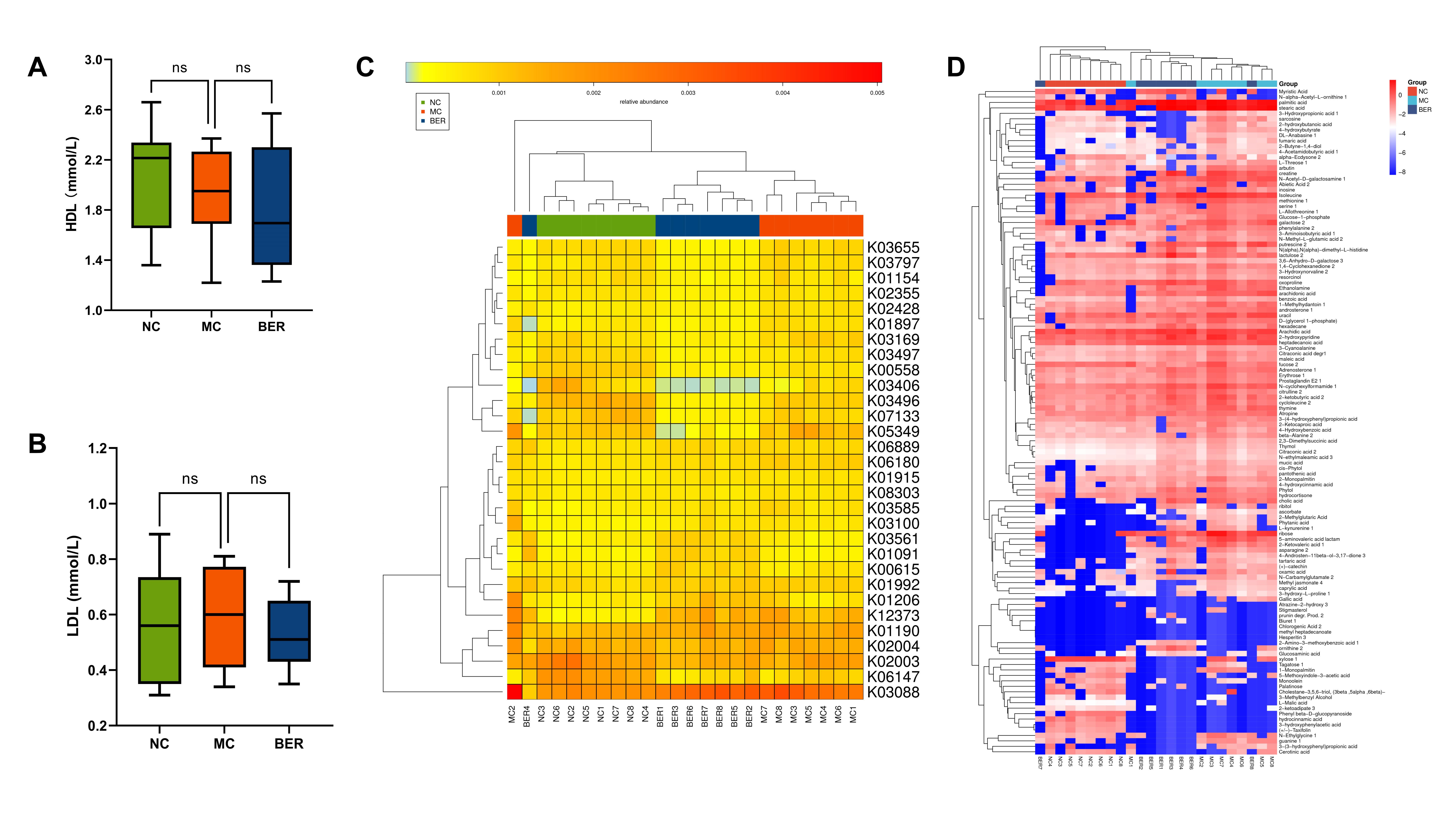

Supplement: Supplementary file 1 [file Image1.JPEG]

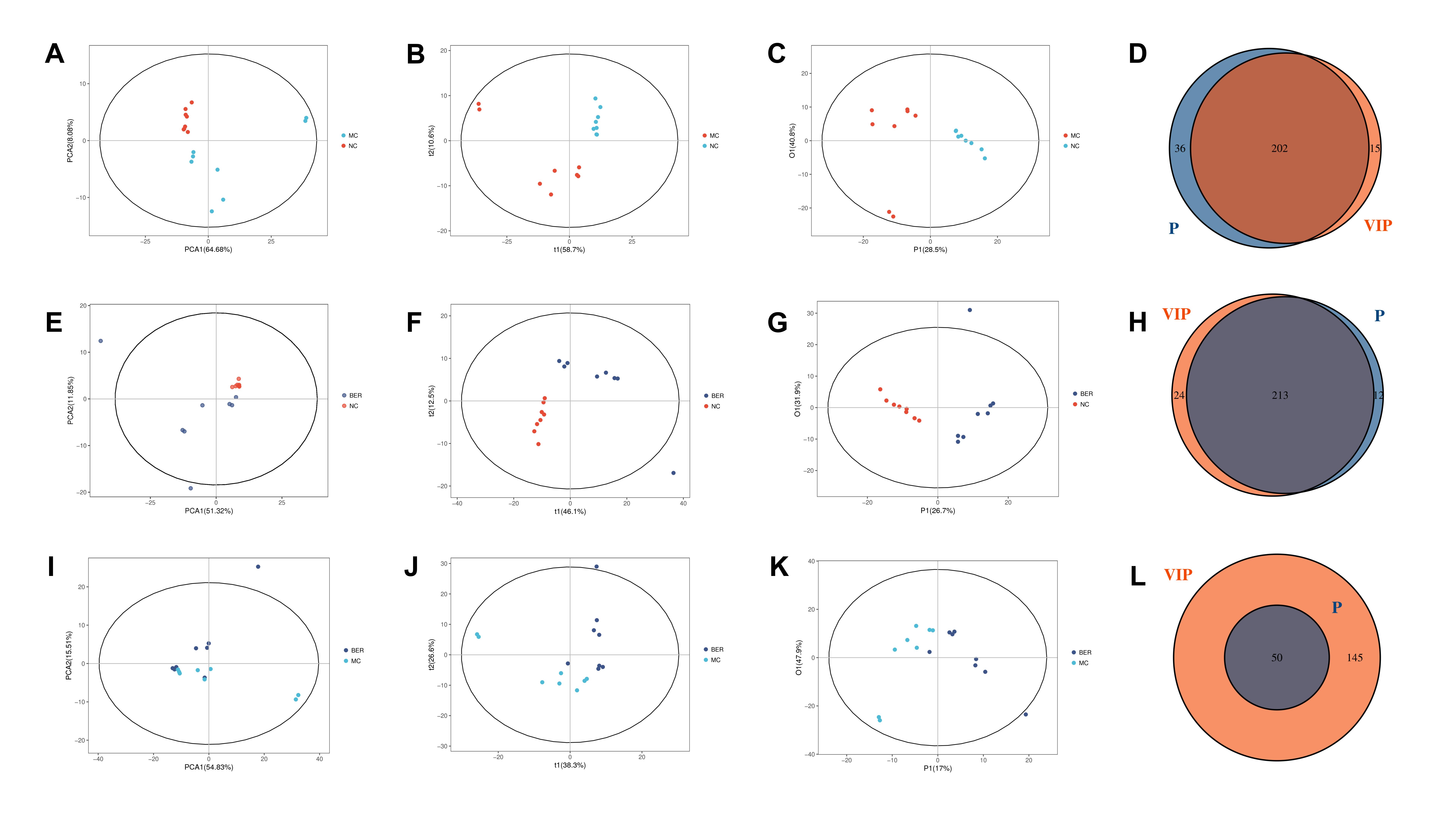

Supplement: Supplementary file 2 [file Image2.JPEG]
