# Supplementary material for: Modulation of Gut Microbiota and Metabolites by Berberine in Treating Mice With Disturbances in Glucose and Lipid Metabolism
Source: Front Pharmacol. 2022 Jun 3;13:870407. doi: 10.3389/fphar.2022.870407 (PMC9204213; doi:10.3389/fphar.2022.870407)
Supplement: Supplementary file 3 [file Table2.DOCX]

**Table 1.** The median and quartile of the Shannon-Weiner index and the Simpson index in each group

|  | **NC group** | **MC group** | | **BER group** | |
| --- | --- | --- | --- | --- | --- |
| **Shannon-weiner index** | 3.16（3.15，3.24） | | 2.38（2.36，2.76） | | 1.93（1.69，1.98） |
| **Simpson index** | 0.94（0.94，0.94） | | 0.83（0.82，0.88） | | 0.75（0.7，0.77） |
